# Risk factors and survival analysis of patients with distant metastatic follicular thyroid cancer

**DOI:** 10.3389/fendo.2025.1748243

**Published:** 2026-01-14

**Authors:** Na Han, Zengmei Si, Guoqiang Wang, Guohua Qin, Chenghui Lu, Xufu Wang

**Affiliations:** Department of Nuclear Medicine, the Affiliated Hospital of Qingdao University, Qingdao, Shandong, China

**Keywords:** cancer-specific survival, distant metastasis, follicular thyroid cancer (FTC), prognosis, risk factors

## Abstract

**Objective:**

We aimed to identify clinicopathological risk factors associated with distant metastasis in follicular thyroid cancer (FTC) and to evaluate prognostic factors influencing survival in distant metastatic FTC patients, thereby providing evidence for risk stratification and personalized treatment strategies.

**Methods:**

In this retrospective study, we enrolled FTC patients who underwent total thyroidectomy, subtotal thyroidectomy, or thyroid lobectomy at the Affiliated Hospital of Qingdao University from January 2014 to December 2021. Eligible patients were divided into 2 groups: the distant metastasis group (DM group) and the group with no evidence of distant metastasis during the study period (NDM group). The DM group was further divided into the survival group and the mortality group at the last follow-up.

**Results:**

In total, 111 patients who underwent thyroid surgery were included. 30 patients (27.03%) had distant metastasis (DM group), and 81 patients (72.97%) had no distant metastasis (NDM group). Multivariate logistic regression analysis indicated that the FTC subtype (odds ratio [OR]: 141.244; 95% confidence interval [CI]: 7.128–2798.802; P = 0.001), the number of lymph node metastases LNMs (OR: 0.028; 95% CI: 0.001–0.563; P = 0.020), T stage (OR: 0.048; 95% CI: 0.003–0.766; P = 0.032) and the type of initial surgery (OR: 175.685; 95% CI: 6.452–4783.472; P = 0.002) were independent risk factors predicting DM. Overall, the 3-year cumulative survival rates of DM patients was 83.0%. Kaplan–Meier survival analysis revealed significant differences in the 3-year survival time according to T stage (P = 0.019).

**Conclusion:**

Widely invasive FTC, lymph node metastasis, T3/T4 stage, and initial total thyroidectomy are independent predictors of distant metastasis in FTC patients. For FTC patients with DM, high T stage may be related to a greater likelihood of mortality.

## 1 Introduction

Follicular thyroid cancer (FTC) is the second most common subtype of differentiated thyroid cancer (DTC), accounting for approximately 10%-15% of all thyroid malignancies (1). In accordance with the World Health Organization (WHO) classification 5th edition of thyroid neoplasms, FTC is categorized into three subtypes: minimally invasive FTC (MIFTC), encapsulated angioinvasive FTC (EAIFTC) and widely invasive FTC (WIFTC) (1). Compared with papillary thyroid cancer (PTC), FTC exhibits a more aggressive biological behavior characterized by hematogenous spread, with distant metastasis (e.g., to the lungs, bones, and brain) being a critical prognostic factor (2). Studies have demonstrated that the 5-year survival rate of FTC patients with distant metastasis significantly decreases (from 98% to 50%-60%), and early detection and intervention of metastatic lesions are crucial for improving outcomes (3). However, owing to the relative rarity of FTC cases, many published studies have often analyzed FTC and PTC collectively, despite their distinct biological behaviors, which has led to potentially inaccurate conclusions. Furthermore, recent evidence regarding risk factors for distant metastasis in FTC patients remains controversial, particularly regarding the clinical significance of parameters such as age, tumor size, and vascular invasion (4, 5).

Therefore, it is important to analyze the risk factors for distant metastasis in FTC patients and to explore the predictors of survival after metastasis to optimize clinical decision-making and individualized management. The aim of this study was to identify independent risk factors associated with distant metastasis in FTC and to evaluate prognostic factors influencing survival in distant metastatic FTC patients through the retrospective cohort analysis. We expect that these findings provide evidence-based data for the early identification of high-risk patients and for the development of stratified follow-up strategies while supplementing clinical evidence for the prognostic assessment of metastatic FTC.

## 2 Materials and methods

### 2.1 Study population

In this retrospective study, we enrolled FTC patients who underwent total thyroidectomy, subtotal thyroidectomy, or thyroid lobectomy at the Affiliated Hospital of Qingdao University from January 2014 to December 2021. The study was approved by the Institutional Review Board of the Affiliated Hospital of Qingdao University. Informed consent was not obtained because the study was retrospective.

The inclusion criteria were as follows: (1) patients who underwent total thyroidectomy, subtotal thyroidectomy, or thyroid lobectomy with or without neck dissection and who were diagnosed with FTC through pathological examination, and (2) patients with a follow-up at least 24 months after the initial surgery. The exclusion criteria included the following: (1) patients with other malignant tumors and (2) patients lost to follow-up.

Eligible patients were divided into 2 groups: the distant metastasis group (DM group) and the group with no evidence of distant metastasis during the study period (NDM group). The DM group was further divided into a survival group and a mortality group at the last follow-up.

### 2.2 Clinicopathological variables

Data on the main clinicopathological variables, including gender, age at diagnosis, subtype of FTC, maximal tumor size, bilateral or unilateral primary lesion, tumor with extrathyroidal extension (ETE), subtype of FTC, number of lymph node metastases (LNMs), tumor stage, N stage, TNM stage, extent of initial surgery, site of metastasis, time of DM, stimulated Tg (sTg) levels, 131I total dose, and radioiodine therapy (RAIT) and tyrosine kinase inhibitor (TKI) therapy, were collected.

### 2.3 RAIT procedures

The patients achieved the goal thyroid stimulating hormone (TSH) concentration of 30 mU/L after thyroid hormone withdrawal and followed a low-iodine diet for 3–4 weeks. The RAIT dose was referred to as each patient’s specific extent of disease on the basis of the 2015 ATA guidelines’ recommendations. Posttherapeutic whole-body scanning (Rx-WBS) and single-photon emission computed tomography/computed tomography (SPECT/CT) were performed 3–5 days after RAIT. After RAIT, levothyroxine treatment was continued.

### 2.4 Statistical analysis

Continuous variables with a normal distribution are summarized as the means ± standard deviations. Continuous variables without a normal distribution are presented as the medians and interquartile ranges (IQRs). Categorical variables are presented as numbers and percentages. Receiver operating characteristic (ROC) curves were used to determine the optimal cutoff values for maximal tumor size and number of LNMs. Logistic regression was used to determine which variables were associated with distant metastasis. Multicollinearity among the candidate variables was assessed prior to multivariable analysis. Variance inflation factors (VIFs) were calculated; variables with a VIF > 5 were considered redundant and excluded from the logistic regression model to ensure model stability. Cancer-specific survival (CSS) was evaluated using the Kaplan–Meier survival method, and the log-rank test was used to evaluate differences between groups.

## 3 Results

### 3.1 Baseline patient characteristics

**Figure 1:**
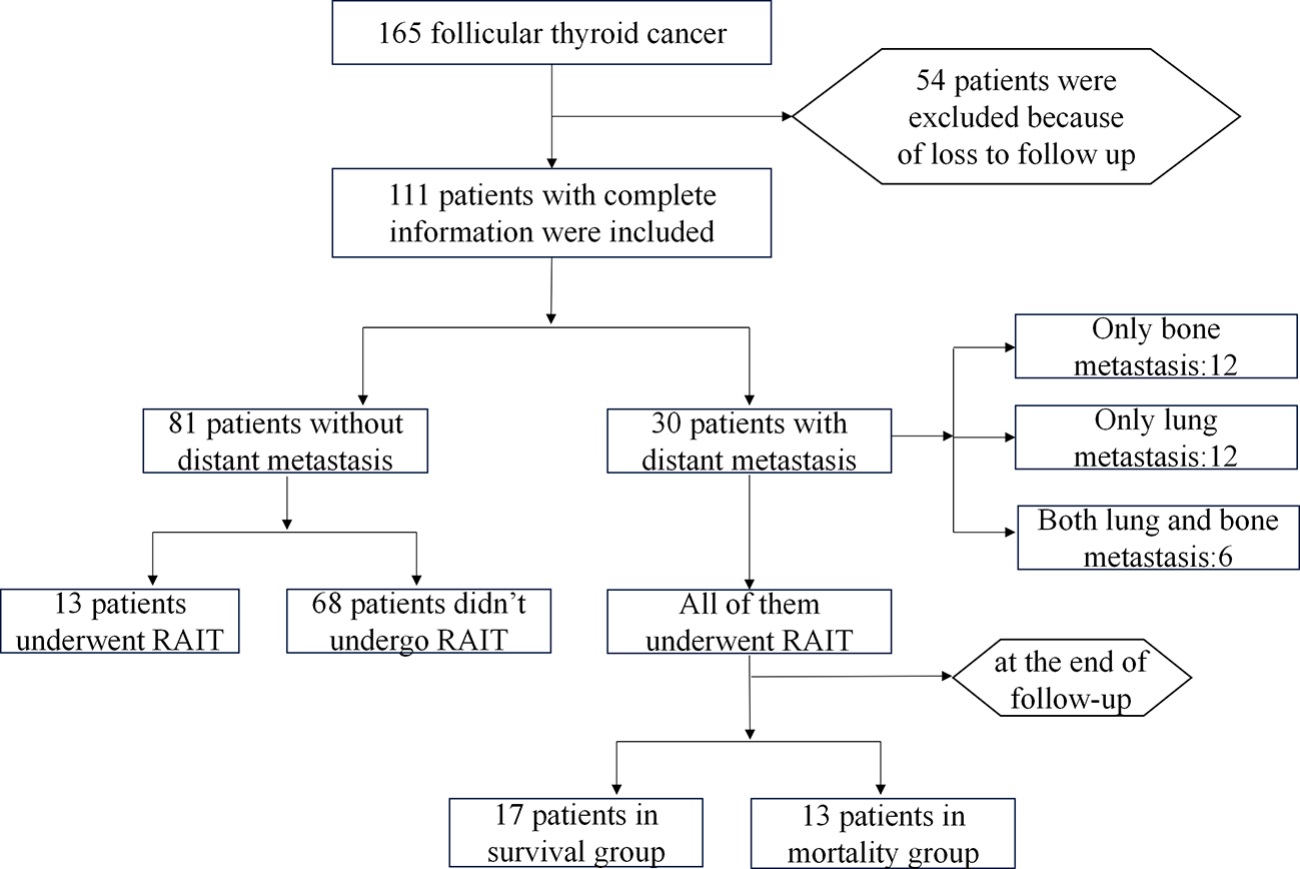
Flowchart of patient inclusion and exclusion in the study.

As shown in **Figure 1**, a total of 111 FTC patients with a mean follow-up duration of 49 months were included. 30 patients (27.03%) developed distant metastasis (DM group), while 81 patients (72.97%) did not (NDM group). In the DM group, 12 patients had isolated bone metastasis, 12 had isolated lung metastasis, and 6 had both bone and lung metastases. The lymph node metastasis (LNM) rate for the entire cohort was 15.3% (17/111). RAIT was performed in 43 patients: all 30 from the DM group and 13 from the NDM group.

ROC curve analysis revealed that the areas under the curve (AUCs) for maximal tumor size and number of LNMs for predicting DM were 0.557 (95% confidence interval [CI]: 0.400–0.713) and 0.620 (95% CI: 0.529–0.710), respectively. The corresponding optimal cutoff values were 6.1 cm and 0.5, respectively. The clinical characteristics of the enrolled FTC patients are presented in **Table 1**.

**Table 1:** Clinical characteristics of enrolled FTC patients.

Characteristics	N (%)	Median (IQR)	Range
Gender			
Male	28 (25.2)		
Female	83 (74.8)		
Age at diagnosis			
<55 y	74 (66.7)	49.0(34.0,60.0)	11.0-76.0
≥55 y	37 (33.3)		
Tumor size (cm)		4.0(3.0,5.6)	0.3-10.0
ETE			
Yes	100 (90.1)		
No	11 (9.9)		
Primary foci			
Unilateral	104 (93.7)		
Bilateral	7 (6.3)		
No. of LNMs		0(0,0)	0-26
T stage			
1	11 (9.9)		
2	25 (22.5)		
3	52 (46.8)		
4	23 (20.8)		
N stage			
N0	94 (84.7)		
N1	17 (15.3)		
M stage			
M1	30 (27.0)		
M0	81 (73.0)		
TNM stage			
1	67 (60.4)		
2	27 (24.3)		
3	2 (1.8)		
4	15 (13.5)		
Subtype			
MIFTC	43 (38.8)		
EAIFTC	34 (30.6)		
WIFTC	34 (30.6)		
Extent of initial surgery			
Total thyroidectomy	37 (33.3)		
Lobectomy+ subtotal thyroidectomy	74 (66.7)		
RAIT			
Yes	43 (38.7)		
No	68 (61.3)		

ETE, extrathyroidal extension; LNM, lymph node metastases; MIFTC, minimally invasive follicular cancer; EAIFTC, encapsulated angioinvasive follicular cancer; WIFTC, widely invasive follicular cancer.

### 3.2 Univariate and multivariate logistic regression analyses for predicting DM in FTC patients

Univariate logistic regression analysis revealed that the FTC subtype, number of LNMs, T stage, and type of initial surgery significantly differed between the DM group and NDM group. Conversely, gender, age at diagnosis, tumor size, primary foci and ETE were not significantly related to DM. However, vascular invasion (VIF = 7.269) and vascular tumor thrombus (VIF = 7.073) were excluded from the univariate analysis because of collinearity with extracapsular extension (ETE). We subsequently analyzed the significantly different factors with multivariate logistic regression analysis. We found that the FTC subtype (odds ratio [OR]: 141.244; 95% confidence interval [CI]: 7.128–2798.802; P = 0.001), number of LNMs (OR: 0.028; 95% CI: 0.001–0.563; P = 0.020), T stage (OR: 0.048; 95% CI: 0.003–0.766; P = 0.032) and type of initial surgery (OR: 175.685; 95% CI: 6.452–4783.472; P = 0.002) were independent risk factors for predicting DM. **Table 2** shows the results of the univariate and multivariate logistic regression analyses.

**Table 2:** Risk factors associated with DM in FTC patients.

Characteristics	Total(N)	Univariate analysis		Multivariate analysis	
		Odds Ratio (95% CI)	P value	Odds Ratio (95% CI)	P value
Gender	111				
Male	28	Reference			
Female	83	0.902 (0.347 – 2.340)	0.832		
Age at diagnosis	111				
<55	74	Reference		Reference	
≥55	37	2.207 (0.929 – 5.239)	0.073	0.242 (0.034 – 1.718)	0.156
Subtype	111				
MIFTC+ EAIFTC	77	Reference		Reference	
WIFTC	34	40.000 (12.241 – 130.712)	< 0.001	141.244 (7.128 – 2798.802)	0.001
Tumor size (cm)	111				
<6.1	80	Reference		Reference	
≥6.1	18	2.758 (0.917 – 8.296)	0.071	1.181 (0.175 – 7.960)	0.865
Unclear	3				
Primary foci	111				
Bilateral	7	Reference			
Unilateral	104	0.468 (0.098 – 2.224)	0.339		
ETE	111				
Yes	100	Reference			
No	11	1.014 (0.250 – 4.105)	0.985		
No. of LNM	111				
≥1	17	Reference		Reference	
0	94	0.189 (0.064 – 0.560)	0.003	0.028 (0.001 – 0.563)	0.020
T stage	111				
T3+T4	75	Reference		Reference	
T1+T2	36	0.162 (0.045 – 0.577)	0.005	0.048 (0.003 – 0.766)	0.032
Type of initial surgery	111				
Lobectomy+ subtotal thyroidectomy	74	Reference		Reference	
Total thyroidectomy	37	7.529 (2.975 – 19.056)	< 0.001	175.685 (6.452 – 4783.472)	0.002

ETE, extrathyroidal extension; LNM, lymph node metastases; MIFTC, minimally invasive follicular cancer; EAIFTC, encapsulated angioinvasive follicular cancer; WIFTC, widely invasive follicular cancer.

### 3.3 Survival analysis in patients with distant metastatic FTC

In the DM group, 17 patients were alive and 13 patients were deceased at the end of follow-up. Regarding metastatic sites, lung metastasis alone was documented in 12 patients (40.0%), bone metastasis alone was documented in 12 patients (40.0%), and concurrent lung and bone involvement was documented in 6 patients (20.0%), with the lung and bone being the most common metastatic sites in FTC patients.

**Figure 2:**
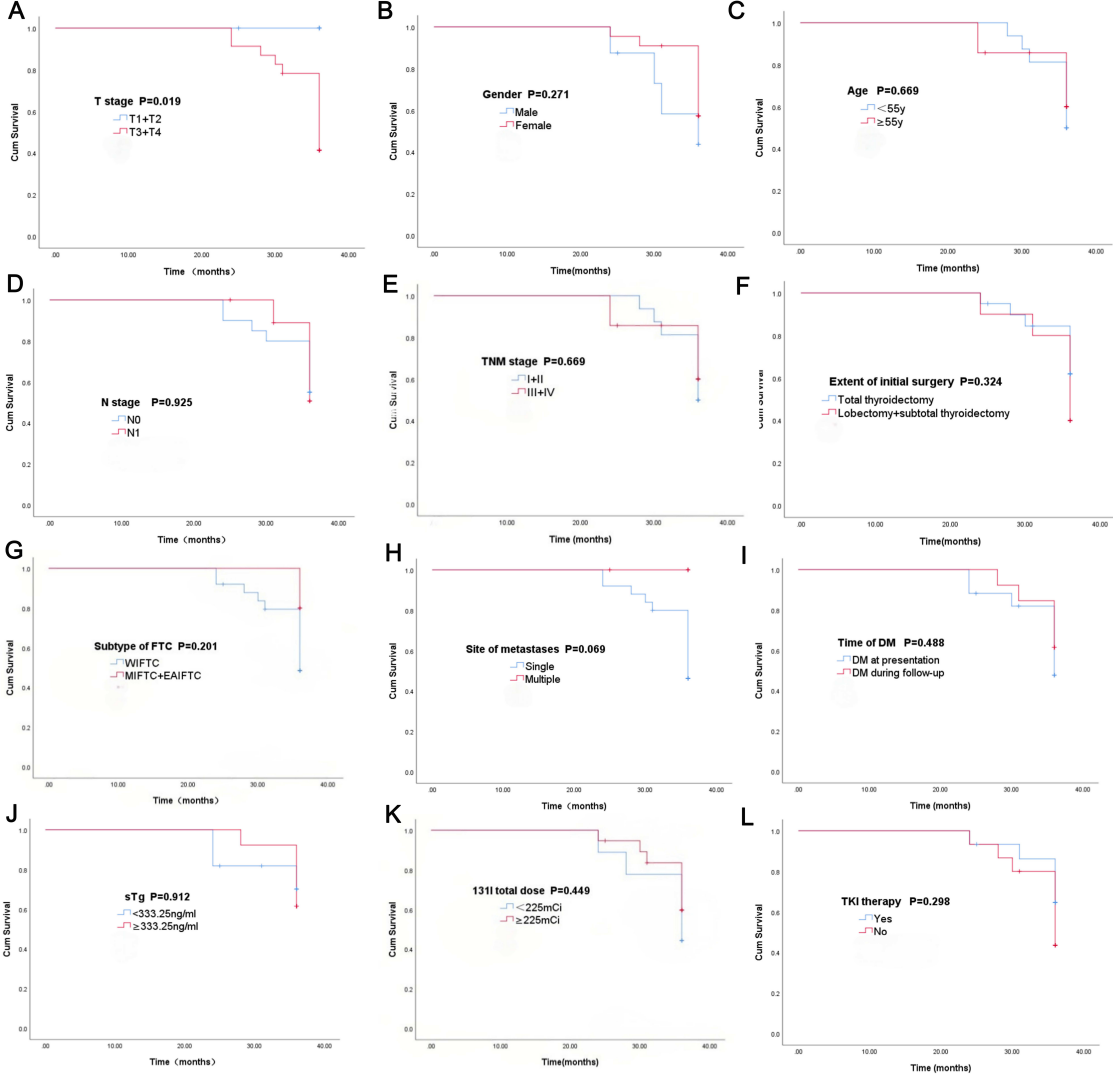
Kaplan–Meier survival plots of the different groups in T stage **(A)**, gender **(B)**, site of metastases **(C)**, N stage **(D)**, TNM stage **(E)**, extent of initial surgery **(F)**, subtype **(G)**, time of DM **(H)**, sTg **(I)**, age **(J)**, 131I total dose **(K)** and TKI therapy **(L)**.

All the DM group patients underwent RAIT at least once. A total of 28/30 patients developed radioactive iodine-refractory DTC (RAIR-DTC). Overall, the 3-year cumulative survival rates of DM patients were 83.0%. Kaplan–Meier survival analysis revealed significant differences in the 3-year survival time according to T stage (P = 0.019), while gender (P = 0.271), age at diagnosis (P = 0.669), N stage (P = 0.925), and TNM stage (P = 0. 669), extent of initial surgery (P = 0.324), subtype of FTC (P = 0.201), the site of metastases (P = 0.069), time of DM (P = 0.488), sTg level (P = 0.912), 131I total dose (P = 0.449), and TKI therapy (P = 0.298) were not significantly different between the different groups. The details are shown in **Figure 2**. The number of patients was too small to conduct multivariate analysis for DM patients.

## 4 Discussion

FTC represents a distinct entity within differentiated thyroid cancer, exhibiting a metastatic behavior markedly distinct from that of PTC (6). In contrast to regional nodal dissemination, FTC preferentially affects distant organs. In this retrospective study, 27.03% of patients presented with distant metastasis, most commonly to bones and lungs, whereas only 15.3% had lymph node involvement. We found that WIFTC (P = 0.001), lymph node metastasis (P = 0.002), high T stage (T3/T4, P = 0.032), and insufficient extent of the initial surgery (P = 0.002) were significant independent predictors of distant metastasis in FTC patients in this cohort.

WIFTC is prone to hematogenous spread because of extensive vascular invasion. We found that the WIFTC subgroup was associated with a 141.244-fold increased risk of DM (OR = 141.244, 95% CI = 7.128–2798.802; P = 0.001). Similarly, previous studies have indicated that the prognosis of WIFTC is significantly poorer than that of minimally invasive FTC (7–10). These findings suggest that the refined classification of pathological subtypes of FTC may enhance the accuracy of DM risk prediction. Doctors can implement intervention measures earlier through refined identification, such as closely monitoring the patient’s condition, promptly detecting signs of distant metastasis, and formulating corresponding treatment strategies to delay disease progression and improve the patient’s survival rate and quality of life.

Lymph node metastasis rarely occurs in patients with FTC compared with PTC, and its presence often indicates greater invasive potential (11). Nguyen et al. (12) reported that the risk of LNM in patients with FTC remains less than 20%, which was consistent with our study (15.3%). The association between T stage and DM risk also corresponds to biological expectations: higher T stages reflect more advanced local invasion, which may increase the likelihood of tumor cells entering systemic circulation (13). We found that a lower T stage (T1+T2) was associated with a reduced risk of DM (OR = 0.048, 95% CI = 0.003–0.766; P = 0.032). Moreover, our results indicated that total thyroidectomy is significantly associated with the risk of distant metastasis, which may reflect its more frequent application in clinical practice for patients with a high tumor burden or poor prognosis. This selection bias might lead to an observed association between the surgical method and the risk of DM.

Survival analysis further revealed that T stage (P = 0.019) significantly impacted survival in DM patients. This finding further imply that, in addition to DM, the extent of local tumor progression (T stage) may modulate survival outcomes by affecting treatment responsiveness or tumor biological characteristics, which is consistent with the finding of previous study (14).

Our study has several limitations. First, this was a single-center study with a relatively small sample size (111 cases). The small sample size may have restricted the statistical power, especially in subgroup analyses. Second, genetic aberration data could not be incorporated into the statistical models because genetic mutational analyses were not conducted in a subset of patients. As Duan et al. demonstrated that such alterations may modulate metastatic risk, their exclusion could have led to the oversight of pivotal predictors (15). Consequently, the sample size should be increased in future investigations through multicenter collaboration to develop a sufficiently powered cohort, validate the prognostic significance of clinicopathological variables such as FTC subtype and T stage, and integrate molecular biomarkers (e.g., RAS and TERT mutations) to construct a more precise risk stratification model.

In summary, widely invasive FTC, LNM, T3/T4 stage, and initial total thyroidectomy are independent predictors of distant metastasis in FTC patients. For FTC patients with DM, high T stage(T3/T4) may be related to a greater likelihood of mortality. Future multicenter investigations with expanded sample sizes that integrate comprehensive histopathological, radiological, and molecular data are expected to increase the precision of FTC metastasis prediction and to refine individualized therapeutic strategies.

## Data Availability

The raw data supporting the conclusions of this article will be made available by the authors, without undue reservation.
